# High third-generation cephalosporin resistant *Enterobacteriaceae* prevalence rate among neonatal infections in Dakar, Senegal

**DOI:** 10.1186/s12879-016-1935-y

**Published:** 2016-10-20

**Authors:** Sebastien Breurec, Coralie Bouchiat, Jean-Marie Sire, Olivier Moquet, Raymond Bercion, Moussa Fafa Cisse, Philippe Glaser, Ousmane Ndiaye, Sidy Ka, Helene Salord, Abdoulaye Seck, Haby Signate Sy, Remy Michel, Benoit Garin

**Affiliations:** 1 Institut Pasteur, Laboratoire de Bactériologie, 36 Avenue Pasteur, BP220 Dakar, Senegal; 2 Centre Hospitalier Universitaire de Pointe-à-Pitre/les Abymes, Laboratoire de Microbiologie clinique et environnementale, BP465, 97159 Pointe-à-Pitre, Guadeloupe France; 3 Faculté de Médecine, Université des Antilles, Campus de Fouillole, BP 145, 97154 Pointe-à-Pitre, Guadeloupe France; 4 Hôpital des Enfants Albert Royer, Laboratoire de Bactériologie, Avenue Cheikh Anta Diop, Dakar, Senegal; 5 Institut Pasteur, Unité de Biologie des Bactéries pathogènes à Gram-positif, 25-28 Rue du Dr Roux, 75015 Paris, France; 6 Département de Pédiatrie, Centre Hospitalier Abass Ndao, BP 15872 Dakar, Senegal; 7 Département de Pédiatrie, Hôpital Principal, 1 avenue Nelson Mandela, BP3006 Dakar, Senegal; 8 Hôpital de la Croix-Rousse, Laboratoire de Bactériologie, 103 Grande rue de la Croix-Rousse, 69317 Lyon, France; 9 Département de Pédiatrie, Hôpital des Enfants Albert Royer, Avenue Cheikh Anta Diop, Dakar, Senegal; 10 Institut Pasteur de Dakar, Unité d’Epidémiologie, 36 Avenue Pasteur, BP220 Dakar, Senegal

**Keywords:** Neonatal infections, Antimicrobial resistance, Third-generation cephalosporin-resistant Enterobacteriaceae, CTX-M-15, Africa

## Abstract

**Background:**

Neonatal infection constitutes one of Senegal’s most important public health problems, with a mortality rate of 41 deaths per 1,000 live births.

**Methods:**

Between January 2007 and March 2008, 242 neonates with suspected infection were recruited at three neonatal intensive care units in three major tertiary care centers in Dakar, the capital of Senegal. Neonatal infections were confirmed by positive bacterial blood or cerebrospinal fluid culture. The microbiological pattern of neonatal infections and the antibiotic susceptibility of the isolates were characterized. In addition, the genetic basis for antibiotic resistance and the genetic background of third-generation cephalosporin-resistant (3GC-R) *Enterobacteriaceae* were studied.

**Results:**

A bacteriological infection was confirmed in 36.4 % (88/242) of neonates: 22.7 % (30/132) during the early-onset and 52.7 % (58/110) during the late-onset periods (*p* > 0.20). Group B *streptococci* accounted for 6.8 % of the 88 collected bacterial isolates, while most of them were *Enterobacteriaceae* (*n* = 69, 78.4 %). Of these, 55/69 (79.7 %) were 3GC-R. The *bla*_CTX-M-15_ allele, the *bla*_SHV_ and the *bla*_TEM_ were highly prevalent (63.5, 65.4 and 53.8 %, respectively), usually associated with *qnr* genes (65.4 %). Clonally related strains of 3GC-R *Klebsiella pneumoniae* and 3GC-R *Enterobacter cloacae*, the two most commonly recovered 3GC-R *Enterobacteriaceae* (48/55), were detected at the three hospitals, underlining the role of cross-transmission in their spread. The overall case fatality rate was 18.6 %.

**Conclusions:**

Measures should be taken to prevent nosocomial infections and the selection of resistant bacteria.

## Background

The fourth Millennium Development Goal, established after the United Nations Millennium Summit in 2000, seeks to decrease the mortality of children under five years of age by two-thirds before 2015 [1]. Sub-Saharan Africa has seen a rapid decline in its under-five mortality rate, with the annual rate of reduction doubling between 1990–2000 and 2000–2013. However, progress in reducing the neonatal mortality rate remains virtually stagnant in this part of the world. The number of neonatal deaths has in fact increased in sub-Saharan Africa, from 944,000 in 1990 to 1,029,000 in 2013 [2].

Neonatal sepsis is defined as a clinical syndrome occurring within the first 28 days of life, manifested by systemic signs of infection, and isolation of a bacterial pathogen from the bloodstream. Neonatal bloodstream infection is associated with significant morbidity, poor long-term outcomes, and a reported mortality rate of 12–20 % [3, 4]. The early-onset period, defined as infection within three days of birth (except for group B *streptococci* (GBS), 0–6 days after birth), classically reflects vertically transmitted infections. Late-onset infections are usually attributed to bacteria acquired from the infant’s surroundings (hospitals or community), *Klebsiella* spp. and *Staphylococcus aureus* being the most frequently recovered [5]. Among hospital born babies, most neonatal infections are hospital-acquired (HA) in developing countries, irrespective of the period of infection. Extended-spectrum beta-lactamase (ESBL)-producing *Klebsiella pneumoniae* is the most frequent HA pathogen [5], along with *Staphylococcus aureus* and *Escherichia coli* [6]. Third-generation cephalosporins (3GC) are commonly used as first-line treatment for neonatal infections in developing countries, resulting in a gradual upward trend in the number of antibiotic resistant isolates, especially among *Enterobacteriaceae* [5].

Senegal is a resource-limited country in equatorial Africa (ranked 163/187 according to the Human Development Index in 2013). Neonatal infection is one of Senegal’s most important public health problems, with a neonatal mortality rate of 21 deaths per 1,000 live births in 2015 [7]. This is four times the rate observed in developed countries [8]. Moreover, there is little data on bacterial resistance patterns in neonatal infections in Senegal or in developing countries [6].

We characterized the bacterial pathogens associated with neonatal infection and their antibiotic susceptibility patterns, in three major tertiary care centers; we also studied the genetic basis for antibiotic resistance and the genetic background of third-generation cephalosporins resistant (3GC-R) *Enterobacteriaceae*.

## Methods

### Patients

From January 2007 to March 2008, neonates (≤28 days) admitted to the neonatal intensive care units in the Hôpital Principal de Dakar (HPD), Hôpital Aristide Le Dantec (HALD) and Hôpital des Enfants Albert Royer (HEAR), were consecutively pre-included if infection was suspected according to the presence of at least two of the following clinical signs: fever (temperature > 37.8 °C) or hypothermia (temperature <35 °C), respiratory signs (apnea, tachypnea (>60 breaths per minute), respiratory distress), neurological signs (hypotonia, weak reflexes, refusal to suck, perturbation of consciousness, convulsions, coma, irritability), digestive signs (vomiting, diarrhea), jaundice (early [<24 h after birth] or prolonged) and cutaneous signs (purpura). One blood culture (standard volume 1–2 ml) was performed before starting antibiotic treatment for each neonate. A lumbar puncture was prescribed except i) in severely ill infants with either cardiovascular or respiratory distress, tense or bulbing anterior fontanelle, the presence of severe thrombocytopenia, or infection around the lumbosacral region [9], ii) in case of parents or legal guardians’s refusal. Neonatal infection was confirmed by a positive blood and/or cerebrospinal fluid culture. Empirical antibiotic therapy was considered inappropriate if the treatment regimen did not include at least one antibiotic that was active *in vitro* against the infecting microorganisms. The cause of death for patients who died during hospitalization was recorded according to the clinician’s assessment. Attributable mortality was defined as neonatal death within three days after the onset of infection.

Neonates were classified into two groups depending on the age at the onset of clinical signs: early-onset period in infants between zero and three days of age (except for GBS, 0–6 days) and late-onset period in infants after three days of age (except for GBS, >6 days).

### Microbiological analysis, antimicrobial susceptibility testing and detection of ESBL

Blood cultures (Hemoline performance diphasic medium, bioMerieux, Marcy l'Etoile, France) were incubated aerobically at 37 °C and observed daily during seven days. In cases of visible microbial growth, blood and chocolate agar plates (Biorad laboratories, Richmond, CA) were inoculated, incubated in a 5 % CO2 atmosphere at 37 °C, and examined after 24 and 48 h. Cerebrospinal fluid (CSF) were collected in sterile containers and processed within 2 h. Schaedler broth with vitamin K3, blood and chocolate agar plates (Biorad laboratories, Richmond, CA) were inoculated, incubated at 37 °C in a 5 % CO2 atmosphere, and examined after 24 and 48 h. API biochemical galleries (API, bioMerieux, Marcy l’Etoile, France) were used for bacterial identification. Growth of *Bacillus*, *Corynebacterium*, and *Micrococcus* species as well as coagulase-negative staphylococci in the blood cultures was considered to be due to contaminants and these cultures were excluded.

Antibacterial drug susceptibility was determined using the disc diffusion method according to the guidelines of the French Society of Microbiology. Production of ESBL in 3GC-R *Enterobacteriaceae* was detected using the double-disc synergy test [10].

### DNA extraction and detection of beta-lactamase and plasmid-mediated quinolone resistance genes

Previously described PCRs were used to screen for carbapenem-hydrolyzing β-lactamase genes (*bla*_VIM_, *bla*_IMP_ and *bla*_KPC_), as well as plasmid-encoded *bla*_CTX-M_, *bla*_AmpC_, *bla*_SHV_, *bla*_TEM_ beta-lactamase genes, the *aac(6′)-Ib* gene, and quinolone resistance *qnr*A/B/S and *qepA* genes [11–13]. All *aac(6′)-Ib* positive strains were further analyzed by digestion of the PCR product with BtsCl (New England Biolabs, Ipswich, MA) to identify the mutated *aac(6’)-Ib-cr* gene, which lacks the BtsCl restriction site present in the wild-type gene [14]. Isolates resistant to cefoxitin were tested for the presence of six families of the plasmid-borne *ampC* gene (FOX, ACC, EBC, MOX, CIT, DHA) [15] and *bla*_CMY-2_.

### Pulsed Field Gel Electrophoresis of Enterobacteriaceae

*Escherichia coli, Klebsiella pneumoniae* and *Enterobacter cloacae* were analyzed by Pulsed Field Gel Electrophoresis (PFGE) as previously described [16].

### Multilocus sequence typing of Group B streptococci

Group *B streptococci* (GBS) were studied by multilocus sequence typing (MLST) using the MLST website for assignments [17].

### Data analysis

Qualitative data were compared using the chi square test and Student’s t test. P values lower than 0.05 were considered to be significant. Statistical analysis was performed using Stata® software 12.0 (Stata Corporation, College Station. Tex.).

## Results

### Patients and outcome

Over the 15-month study period, 242 neonates with suspected infection were consecutively included, among whom 19 (7.9 %) were born at home: 119 (49.2 %) from HPD, 86 (35.5 %) from HEAR and 37 (15.3 %) from HALD. The sex ratio (M/F) was 1.4, the median age 3 days and median weight 2700 g; 54.5 % (132/242) were in the early-onset period.

A bacteriological infection was confirmed in 36.4 % (88/242) of the neonates (66 from HPD, 13 from HEAR and 9 from HALD), among whom 36.4 % (32/88) were premature; 22.7 % (95 % confidence interval (CI): 15.6–29.8 %, 30/132) of those included during the early-onset period and 52.7 % (95 % CI: 48.8–67.2 %, 58/110) of those included during the late-onset period (*p* > 0.20). The positivity rates of blood and CSF cultures were 34.1 % (77/226) and 68.8 % (11/16), respectively. All neonates born at home (*n* = 9) were included during the late-onset period. The median length of hospital stay was 7 days (range 1–30 days).

Neonates presented the following clinical signs: fever (*n* = 15, 17,0 %) or hypothermia (*n* = 20, 22,7 %); respiratory signs including respiratory distress (*n* = 30, 34,1 %), tachypnea (*n* = 26, 29,5 %), apnoea (*n* = 4, 4,5 %); neurologic signs including refusal to suck (*n* = 39, 44,3 %), hypotonia (*n* = 29, 33,0 %), weak reflexes (*n* = 12, 13,6 %) and perturbation of consciousness (*n* = 9, 10,2 %); digestive signs including vomiting (*n* = 5, 5,7 %) and diarrhea (*n* = 2, 2,3 %); jaundice (early (*n* = 18, 20,5 %), prolonged (*n* = 7, 8,0 %)); purpura (*n* = 2, 2,3 %); cardiovascular collapse (shock) (*n* = 3, 3,4 %). No significant difference was found between neonates with sepsis and those with meningitis (*p* > 0.20).

The outcome was known for 86 out of the 88 infected neonates: 18.6 % (16/86) [10.4–26.8 % 95 % CI] died, with no statistical difference (*p* = 0.39) between early-onset (12/55, 21.8 %) and late-onset (4/31, 12.9 %). Lethality associated with sepsis was 17.3 % (13/75, 95 % CI: 8.7–25.9 %,), and that associated with meningitis, 27.3 % (3/11, 95 % CI: 1–53.6 %).

### Bacterial strains, antibiotic susceptibility and antibiotic treatment

Among the 88 strains associated with neonatal infections, 69 (78.4 %) were *Enterobacteriaceae*, of which 66 were isolated from blood cultures (Table 1). Fifty-five *Enterobacteriaceae* strains (79.7 %, 55/69) were resistant to 3GC (33 *K. pneumoniae,* 15 *E. cloacae*, 4 *E. coli*, 2 *Serratia liquefasciens* and 1 *E. aerogenes*). All 3GC-R *Enterobacteriaceae* isolates were resistant to all beta-lactams tested, except for cefoxitin (31.9 % resistance, 22/69), and all were susceptible to imipenem. In addition, *Enterobacteriaceae* isolates had high rates of resistance to cotrimoxazole (79.8 %), gentamicin (79.8 %), tobramycin (76.8 %), and ciprofloxacin (66.7 %), a moderate rate of susceptibility to amikacin (52.2 %), and a high rate of susceptibility to fosfomycin (98.6 %).

**Table 1:** Distribution of 88 bacterial species by specimen (77 from blood and 11 from cerebrospinal fluid) isolated during the neonatal period

Specimen	Bacterial group	Bacterial species	Neonatal period	
			Early-onset period N	Late-onset period N
Blood	*Enterobacteriaceae*	*Klebsiella pneumoniae*	15	24
		*Enterobacter cloacae*	6	7
		*Enterobacter aerogenes*	1	0
		*Escherichia coli*	2	6
		*Cirobacter koseri*	0	1
		*Serratia liquefacienx*	1	1
		*Serratia marcescens*	0	2
	Gram-negative non-*Enterobacteriaceae*	*Pseudomonas aeruginosa*	1	1
		*Acinetobacter baumanii*	1	0
	Gram-positive cocci	Group B *streptococci*	2	0
		*Staphylococcus aureus*	1	1
		*Enterococcus faecium*	0	3
		*Enterococcus faecalis*	0	1
Cerebrospinal fluid	*Enterobacteriacae*	*Enterobacter cloacae*	0	1
		*Escherichia coli*	0	1
		*Morganella morganii*	0	1
	Gram-negative non-*Enterobacteriaceae*	*Pseudomonas aeruginosa*	0	2
	Gram-positive cocci	Group B *streptococci*	0	4
		*Streptococcus pneumoniae*	0	2

The Gram-negative non-*Enterobacteriaceae* (5/88, 5.7 %) were *Pseudomonas aeruginosa* (*n* = 4) and *Acinetobacter baumanii* (*n* = 1). These isolates were collected from blood (3/77, 3.9 %) and CSF (2/11, 18.2 %) cultures (Table 1). One *P. aeruginosa* isolate was imipenem and levofloxacin resistant whereas the *A. baumanii* isolate was resistant to ceftazidime.

GBS was only isolated from 6.7 % (2/30) cases in the early-onset and 6.9 % (4/58) cases in the late-onset periods. GBS was found in 2.6 % (2/77) of positive blood cultures and 36.4 % (4/11) of positive CSF cultures (Table 1). During the early-onset period, one GBS infected case died with sepsis. All isolates were ampicillin and erythromycin susceptible. *Streptococcus pneumoniae* was isolated from CSF (2/11, 18.2 %); both isolates were oxacillin susceptible. The two *S. pneumoniae* infected cases died during the early-onset period. *Enterococcus faecalis*/*faecium* (4/77, 5.2 %) and *Staphylococcus aureus* (2/77, 2.6 %) were isolated from blood during the late-onset period except for one *S. aureus* (Table 1). Both *S. aureus* isolates were methicillin susceptible.

The empirical antibiotic treatment consisted of a combination of two or three antibiotics. The most frequently prescribed antibiotics were gentamicin (100 %), 3GC (78.8 %) and amoxicillin (31.8 %). The rate of inadequate antibiotic treatment was 65.9 % (58/88), mainly due to *Enterobacteriaceae* resistant to both 3GC and gentamicin (79.7 %, 55/69).

### Antibiotic resistance determinants in 3GC-resistant (3GC-R) Enterobacteriaceae strains

All 3GC-R *Enterobacteriaceae* were tested for resistance genes except for one *E. aerogenes* and two *Serratia liquefasciens* strains: 69.2 % (36/52) were positive for *bla*_CTX-M_ group 1 (*n* = 33) and group 8 (*n* = 3), 65.4 % (34/52) for *bla*_SHV_, 53.8 % (28/52) for *bla*_TEM_ and 3.8 % (2/52) for *ampC* (CMY-2, DHA and CIT positives). All *bla*_CTX-M_ group 1 were assigned to the *bla*_CTX-M-15_ allele. The *aac(6’)-Ib* gene was detected in 41 (78.8 %) of the 52 isolates, of which 34 (82.9 %) were *aac(6’)-Ib-cr*. Of the 34 *qnr*-positive strains (65.4 %), 31 were *qnrB*, three were *qnrS,* and *qnrA* and *qepA* were not detected (Table 2).

**Table 2:** Antibiotic resistance genes and Pulsed Field Gel Electrophoresis profiles of 52 *E. coli*, *K. pneumoniae* and *Enterobacter cloacae* isolates resistant to third-generation cephalosporins

Bacteria species (N)	*bla*_*TEM*_	*bla*_*SHV*_	*bla*_*CTX-M-15*_	*bla*_*CTX-M*_ *group 8*	*bla*_*AmpC*_	*aac-(6)’-Ib-cr*	*qnrB*	*qnrS*	PFGE profiles
*Klebsiella pneumoniae* (33)	18	30	24	3	1 (CIT)	23	21	3	1 to 12
*Enterobacter cloacae* (15)	9	2	8	0	1 (CMY-2, DHA)	8	10	0	1 to 10
*Escherichia coli* (4)	1	2	1	0	0	3	3	0	1 to 4

*PFGE* Pulsed Field Gel Electrophoresis

### Pulsed-field Gel Electrophoresis of 3GC-R Enterobacteriaceae

The 33 *K. pneumoniae* isolates grouped into five main PFGE clusters (3 to 11 isolates) in Pulsed-field Gel Electrophoresis (PFGE) analysis. The 15 *E. cloacae* isolates yielded 10 distinct PFGE patterns, with six strains belonging to a single cluster. These six isolates were isolated in all the three hospitals. Each of the four *Escherichia coli* isolates displayed a distinct PFGE pattern (Table 2).

### MLST of GBS

Five of the six isolates were investigated: ST17 was isolated from two CSF samples from different hospitals, and ST23 (*n* = 2) and ST26 (*n* = 1) from three blood samples.

## Discussion

Blood culture remains the “gold standard” for the detection of neonatal sepsis, highlighting the importance of a high index of suspicion based on clinical and laboratory parameters long before the blood culture results are known. Our study included 242 neonates with suspected neonatal infections. The frequency of neonatal sepsis (34.1 %, 77/226) was consistent with rates reported in other African and Asian developing countries (34.8–45.9 %) [18–20]. However, negative blood culture does not rule out a bacterial infection, as obligate anaerobes were not investigated in our study. In addition, coagulase-negative *staphylococci, Bacillus* spp.*, Corynebacterium* spp. and *Micrococcus* spp. were excluded from this study due to the lack of two successive blood cultures, but it is crucial to recognize that they can also represent a true cause of late-onset infection. A lumbar puncture was performed only in 16 neonates with suspected neonatal infection. Disruption of African hospitals, combined with refusal by children’s parents or legal guardians due probably to a cultural preexisting belief that this medical procedure will cause death [21], lead to a probable underestimate of the true prevalence of meningitis in our studied population. The overall lethality rate was 18.6 %. This value was in the range of reported values in developing countries [5], including Senegal (19.5 % in 1999) [22].

The pattern of pathogens associated with neonatal infections was quite different from that observed in developed countries were GBS and *E. coli* are predominant [9]. GBS and *E. coli* were responsible of 6.8 and 9.1 % of neonatal infection in the current study, respectively. These data are in the range of reported values in Sub-saharan Africa, 2–8 % of cases for GBS and 5–16 % for *E. coli* [23]. The microorganisms most frequently recovered were *Enterobacteriaceae* (78.4 %), for both early and late-onset infections, most of them being resistant to 3GC [5]. This indicates that hospital environmental and human floras are reservoirs of ESBL-*Enterobacteriaceae* and are potential sources of colonization and infection in newborns during delivery and the post-natal period*.* The usual conditions prevailing in hospital settings in low-income countries may favor horizontal transmission of multiresistant bacteria [24]. They probably act as a barrier for GBS and *E. coli* transmission, which could explain their low detection rates among infected neonates. As a result, the majority of early-onset infections may be more hospital acquired, than vertically transmitted. Of note, pregnant women may represent a substantial source of multiresistant *Enterobacteriaceae* for vertical transmission to neonates as highlighted by the high prevalence of ESBL-E among pregnant women from Madagascar [24]. Further studies are needed to differentiate maternally-acquired from hospital-acquired routes of transmission to implement improved preventive strategies in these settings.

Among *Enterobacteriaceae*, 79.8 % were 3GC-R, as previously described in developing countries [5]. Then, a significant proportion of the causal bacteria was not treatable neither by the recommended first-line regimen (ampicillin and gentamicin) [25] nor by alternative C3G treatment. Thus, carbapenems are regarded as the ultimate treatment option. However, these antimicrobials are expensive and unaffordable for the vast majority of the population in these settings, making these bacteria difficult to treat. In South-East Asia, many newborn babies in hospitals are now treated with carbapenems as first-line treatment for sepsis or suspected sepsis. However, carbapenems should not be prescribed as empiric therapy as highlighted by the emergence of resistant untreatable carbapenem-resistant *Enterobacteriaceae* infections associated with high mortality in intensive care units [26]. The alarming use of 3GC observed in our study, contrary to recommendations of antibiotic use [25], has contributed to selection pressure that has provided a competitive advantage for C3G resistant strains. Quality-assured basic microbiology services and routine bacterial resistance surveillance are urgently needed in developing countries in order to reduce antibiotic selection pressure [27]. In addition, all necessary measures for preventing hospital-acquired infections must be implemented. These include standard hygiene practices, especially adherence to hand hygiene policies, which are the cornerstone for preventing the transmission of multidrug-resistant bacteria [27]. Indeed, cross-transmission plays a major role, as suggested by the fact that neonates from the same department shared clonally related strains. This was true for the *K. pneumoniae* and *E. cloacae* strains, which are known to be highly transmissible in hospital settings, especially in intensive care units [26]. In addition, blood culture is time-consuming. Up to 6 days are usually required before antimicrobial susceptibility testing results of bacterial pathogens are available. This delay often causes an inappropriate empiric therapy but also a prolonged length of hospital stay, which favor the horizontal transfer of multidrug resistant strains. Neonates in different hospitals also shared clonally related strains which suggests that there was cross-transmission events between the three health care centers, probably by transfer of infected or colonized patients.

3GC-R resistance in *Enterobacteriaceae* is mainly mediated by ESBL genes located on mobile genetic elements such as plasmids [28]. In our study conducted in 2007–2008, *bla*_CTX-M_ group 1 or *bla*_SHV_ was detected in most of the strains (69.5 %), highlighting the *bla*_TEM_/*bla*_SHV_ to *bla*_CTX-M-15_ shift in the 2000s_,_ observed in Dakar [10, 29] and worldwide [30]. These genes were frequently associated with *qnr* and *aac(6’)-Ib-cr* genes. Frequent association of *bla*_CTXM-15_, *qnrB* and *aac(6’)-Ib-cr* genes has been described in *Enterobacteriaceae* isolates in North, West, Central and East Africa, consistent with the hypothesis that these resistance-determinant genes are carried on the same plasmid [10, 29, 31]. Such an accumulation of resistance genes on a single plasmid prone to horizontal transmission is problematic, especially in countries with inadequate health care systems. Early and appropriate antibiotic treatment is the cornerstone of neonatal infection management [32]. This is of great concern, as 65.9 % of neonates did not receive an appropriate empiric therapy in our study. The emergence of carbapenemases, which has been described in Senegal [33] but was not found in our study, could be a serious challenge for infection control and antibiotic therapy in the future.

In our study, there were only six cases of mother-to-child transmission of GBS. Two isolates, both found in infants with meningitis, were assigned to the ST17 hypervirulent lineage. This ST is known to be associated with neonatal disease and correlated with serotype III [34, 35] which is predominant in meningitis or invasive infections [34]. In 2007, a study conducted in Dakar on healthy pregnant Senegalese women showed a GBS positive vaginal prevalence rate of 20 %. The three STs observed in our study were all major clones in the previous study; ST17, ST23 and ST26, representing 13.3, 14.7 and 20.0 %, respectively, of all GBS in cases of vaginal carriage [36]. Given the resources required for a screening-based approach to guide intrapartum antibiotic prophylaxis, a risk factor-based approach with antibiotics prescribed during labor for women showing risk factors of early-onset GBS disease such as chorioamnionitis, prolonged rupture of membrane, or preterm delivery, [37] is likely to be more applicable.

## Conclusions

These data are of particular importance because of the difficulty to carry out such studies in countries with inadequate health care systems, and the lack of data on pathogens associated with neonatal infections and their antibiotic susceptibilities. 3GC-R *Enterobacteriaceae* were highly prevalent and GBS were detected. All necessary measures should be taken in African hospitals to prevent nosocomial infections and the selection of resistant bacteria by implementing efficient nosocomial infection surveillance programs. Standard hygiene and especially hand hygiene policies need to be reinforced to prevent transmission of multidrug-resistant bacteria.

## Abbreviations

3GC: Third-generation cephalosporins
3GC-R: Third-generation cephalosporin resistant
CSF: Cerebrospinal fluid
ESBL: Extended-spectrum beta-lactamase
GBS: Group B *streptococci*
HA: Hospital-acquired
HALD: Hôpital Aristide Le Dantec
HEAR: Hôpital des Enfants Albert Royer
HPD: Hôpital Principal de Dakar
PFGE: Pulsed-field Gel Electrophoresis
MLST: Multilocus sequence typing

## References

[1] Seale AC, Mwaniki M, Newton CR, Berkley JA (2009). Maternal and early onset neonatal bacterial sepsis: burden and strategies for prevention in sub-Saharan Africa. Lancet Infect Dis.

[2] Healthy Newborn Network Database. http://www.healthynewbornnetwork.org/countries/. Accessed 19 Oct 2016.

[3] Tsai MH, Hsu JF, Chu SM, Lien R, Huang HR, Chiang MC, Fu RH, Lee CW, Huang YC (2014). Incidence, clinical characteristics and risk factors for adverse outcome in neonates with late-onset sepsis. Pediatr Infect Dis J.

[4] Mitha A, Foix-L'Helias L, Arnaud C, Marret S, Vieux R, Aujard Y, Thiriez G, Larroque B, Cambonie G, Burguet A (2013). Neonatal infection and 5-year neurodevelopmental outcome of very preterm infants. Pediatrics.

[5] Zaidi AK, Huskins WC, Thaver D, Bhutta ZA, Abbas Z, Goldmann DA (2005). Hospital-acquired neonatal infections in developing countries. Lancet.

[6] Huynh BT, Padget M, Garin B, Herindrainy P, Kermorvant-Duchemin E, Watier L, Guillemot D, Delarocque-Astagneau E (2015). Burden of bacterial resistance among neonatal infections in low income countries: how convincing is the epidemiological evidence?. BMC Infect Dis.

[7] UN Inter-agency Group for Child Mortality Estimation. http://data.unicef.org/wpcontent/uploads/2015/12/IGME-report-2015-child-mortality-final_236.pdf. Accessed 19 Oct 2016.

[8] Lawn JE, Cousens S, Zupan J, Lancet Neonatal Survival Steering T (2005). 4 million neonatal deaths: when? Where? Why?. Lancet.

[9] Simonsen KA, Anderson-Berry AL, Delair SF, Davies HD (2014). Early-onset neonatal sepsis. Clin Microbiol Rev.

[10] Harrois D, Breurec S, Seck A, Delaune A, Le Hello S, Pardos de la Gandara M, Sontag L, Perrier-Gros-Claude JD, Sire JM, Garin B (2014). Prevalence and characterization of extended-spectrum beta-lactamase-producing clinical *Salmonella enterica* isolates in Dakar, Senegal, from 1999 to 2009. Clin Microbiol Infect.

[11] Guessennd N, Bremont S, Gbonon V, Kacou-Ndouba A, Ekaza E, Lambert T, Dosso M, Courvalin P (2008). Qnr-type quinolone resistance in extended-spectrum beta-lactamase producing enterobacteria in Abidjan, Ivory Coast. Pathol Biol.

[12] Arlet G, Rouveau M, Philippon A (1997). Substitution of alanine for aspartate at position 179 in the SHV-6 extended-spectrum beta-lactamase. FEMS Microbiol Lett.

[13] Yamane K, Wachino J, Suzuki S, Arakawa Y (2008). Plasmid-mediated *qepA* gene among *Escherichia coli* clinical isolates from Japan. Antimicrob Agents Chemother.

[14] Park CH, Robicsek A, Jacoby GA, Sahm D, Hooper DC (2006). Prevalence in the United States of *aac(6')-Ib-cr* encoding a ciprofloxacin-modifying enzyme. Antimicrob Agents Chemother.

[15] Perez-Perez FJ, Hanson ND (2002). Detection of plasmid-mediated AmpC beta-lactamase genes in clinical isolates by using multiplex PCR. J Clin Microbiol.

[16] Shi ZY, Liu PY, Lau YJ, Lin YH, Hu BS (1997). Use of pulsed-field gel electrophoresis to investigate an outbreak of *Serratia marcescens*. J Clin Microbiol.

[17] Jones N, Bohnsack JF, Takahashi S, Oliver KA, Chan MS, Kunst F, Glaser P, Rusniok C, Crook DW, Harding RM (2003). Multilocus sequence typing system for group B *Streptococcus*. J Clin Microbiol.

[18] Shehab El-Din EM, El-Sokkary MM, Bassiouny MR, Hassan R (2015). Epidemiology of neonatal sepsis and implicated pathogens: a study from Egypt. Biomed Res Int.

[19] Mugalu J, Nakakeeto MK, Kiguli S, Kaddu-Mulindwa DH (2006). Aetiology, risk factors and immediate outcome of bacteriologically confirmed neonatal septicaemia in Mulago hospital, Uganda. Afr Health Sci.

[20] Meremikwu MM, Nwachukwu CE, Asuquo AE, Okebe JU, Utsalo SJ (2005). Bacterial isolates from blood cultures of children with suspected septicaemia in Calabar, Nigeria. BMC Infect Dis.

[21] Thakur KT, Mateyo K, Hachaambwa L, Kayamba V, Mallewa M, Mallewa J, Nwazor EO, Lawal T, Mallum CB, Atadzhanov M (2015). Lumbar puncture refusal in sub-Saharan Africa: A call for further understanding and intervention. Neurology.

[22] Cisse C, Mbengue-Diop R, Moubarek M, Nidaye O, Dotou C, Boye CS, Kuakuvi NK, Diadhou F (2001). Infections bactériennes néonatales au CHU de Dakar. Gynecol Obstet Fertil.

[23] Zea-Vera A, Ochoa TJ (2015). Challenges in the diagnosis and management of neonatal sepsis. J Trop Pediatr.

[24] Chereau F, Herindrainy P, Garin B, Huynh BT, Randrianirina F, Padget M, Piola P, Guillemot D, Delarocque-Astagneau E (2015). Colonization of extended-spectrum-beta-lactamase- and NDM-1-producing *Enterobacteriaceae* among pregnant women in the community in a low-income country: a potential reservoir for transmission of multiresistant E*nterobacteriaceae* to neonates. Antimicrob Agents Chemother.

[25] Russell AB, Sharland M, Heath PT (2012). Improving antibiotic prescribing in neonatal units: time to act. Arch Dis Child Fetal Neonatal Ed.

[26] Pena C, Pujol M, Ricart A, Ardanuy C, Ayats J, Linares J, Garrigosa F, Ariza J, Gudiol F (1997). Risk factors for faecal carriage of *Klebsiella pneumoniae* producing extended spectrum beta-lactamase in the intensive care unit. J Hosp Infect.

[27] Woerther PL, Angebault C, Jacquier H, Hugede HC, Janssens AC, Sayadi S, El Mniai A, Armand-Lefevre L, Ruppe E, Barbier F (2011). Massive increase, spread, and exchange of extended spectrum beta-lactamase-encoding genes among intestinal *Enterobacteriaceae* in hospitalized children with severe acute malnutrition in Niger. Clin Infect Dis.

[28] Pfeifer Y, Cullik A, Witte W (2010). Resistance to cephalosporins and carbapenems in Gram-negative bacterial pathogens. Int J Med Microbiol.

[29] Breurec S, Guessennd N, Timinouni M, Le TA, Cao V, Ngandjio A, Randrianirina F, Thiberge JM, Kinana A, Dufougeray A (2013). *Klebsiella pneumoniae* resistant to third-generation cephalosporins in five African and two Vietnamese major towns: multiclonal population structure with two major international clonal groups, CG15 and CG258. Clin Microbiol Infect.

[30] Laxminarayan R, Duse A, Wattal C, Zaidi AK, Wertheim HF, Sumpradit N, Vlieghe E, Hara GL, Gould IM, Goossens H (2013). Antibiotic resistance-the need for global solutions. Lancet Infect Dis.

[31] Rafai C, Frank T, Manirakiza A, Gaudeuille A, Mbecko JR, Nghario L, Serdouma E, Tekpa B, Garin B, Breurec S (2015). Dissemination of IncF-type plasmids in multiresistant CTX-M-15-producing *Enterobacteriaceae* isolates from surgical-site infections in Bangui, Central African Republic. BMC Microbiol.

[32] Tsai MH, Chu SM, Hsu JF, Lien R, Huang HR, Chiang MC, Fu RH, Lee CW, Huang YC (2015). Breakthrough bacteremia in the neonatal intensive care unit: incidence, risk factors, and attributable mortality. Am J Infect Control.

[33] Moquet O, Bouchiat C, Kinana A, Seck A, Arouna O, Bercion R, Breurec S, Garin B (2011). Class D OXA-48 carbapenemase in multidrug-resistant enterobacteria, Senegal. Emerg Infect Dis.

[34] Brzychczy-Wloch M, Gosiewski T, Bulanda M (2014). Multilocus sequence types of invasive and colonizing neonatal group B streptococci in Poland. Med Princ Pract.

[35] Le Doare K, Heath PT (2013). An overview of global GBS epidemiology. Vaccine.

[36] Brochet M, Couve E, Bercion R, Sire JM, Glaser P (2009). Population structure of human isolates of *Streptococcus agalactiae* from Dakar and Bangui. J Clin Microbiol.

[37] Mukhopadhyay S, Puopolo KM (2012). Risk assessment in neonatal early onset sepsis. Semin Perinatol.

